# Gender dimensions of the impact of HIV/AIDS on stunting in children under five years in Zimbabwe

**DOI:** 10.1186/s12889-021-11410-7

**Published:** 2021-07-27

**Authors:** Lesley Macheka, George Kembo, Terrence Kairiza

**Affiliations:** 1 Centre for Innovation and Technology Transfer, Marondera University of Agricultural Sciences and Technology, P. O. Box 35, Marondera, Zimbabwe; 2 Food and Nutrition Council of Zimbabwe, 1574 Alpes Road, Hatcliffe, Harare, Zimbabwe; 3 Department of Economics, Bindura University of Science Education, P. Bag 1020, Bindura, Zimbabwe

**Keywords:** HIV/AIDS, Gender, Nutrition security, Child stunting, Zimbabwe

## Abstract

**Background:**

HIV/AIDS can have a disastrous effect on household food and nutrition security outcomes such as stunting in children under 5. However, stunting and HIV/AIDS are highly gendered phenomena that need to be explored in order to get an in-depth understanding of the interrelationship. This study was therefore aimed at investigating gender dimensions of the impact of HIV/AIDS on stunting in children under 5 years in Zimbabwe.

**Methods:**

The study uses a large scale nationally representative cross-sectional dataset of 13,854 Zimbabwean households for the year 2019. To test hypothesis 1, the study employs binary choice models (Probit and Logit) since the outcome variable household HIV/AIDS status is dichotomous. To test hypothesis 2 and 3, the study employs the Propensity Score Matching (PSM) approach to circumvent the self-selection problem in the creation of treatment and control groups for households affected by HIV/AIDS and those that are not.

**Results:**

The results revealed that household HIV/AIDS status is independent of the gender of household head. On the other hand, the results for the PSM estimates show that the probability of the household having a stunted child under 5 years is higher for households with an HIV positive member compared to those without. In addition, female headed households with an HIV positive member are more likely to have a stunted child under 5 years compared to male headed households under similar circumstances.

**Conclusion:**

Overall, the results provide evidence of a higher risk of stunting among children from households affected by HIV/AIDS. The study offers three major findings. Firstly, the study finds no significant association between gender of the household head and household HIV/AIDS status. Secondly, households that have at least one HIV positive member are more likely to have a stunted child under 5 years. Lastly, female headed households with at least one HIV positive member are more likely to have a stunted child under 5 years compared to male headed household with similar HIV/AIDS status. The findings have important policy implications towards improved integration of HIV/AIDS status, household head gender and child nutrition services in affected households.

## Background

Stunting in children under 5 years is an obstinate motif bedevilling the frail public health systems in sub-Saharan Africa [1]. At least 90% of stunted children under 5 years in the world live in Africa and Asia [2]. The under 5 years old age group is particularly vulnerable since stunting poses permanent negative effects on the physical, cognitive, and social status of the concerned child, concomitantly curtailing economic potential in later life [3, 4]. Efforts to curtail the high prevalence of stunting in Sub-Saharan Africa are confounded by intervening conditions such as the high prevalence of Acquired Immunodeficiency Syndrome (AIDS) / Human Immunodeficiency Virus (HIV) which imposes heterogenic vulnerability to food and nutrition insecurity between households that are affected and those that are not [5].

Households with people living with HIV (PLWHIV) are more likely to be food and nutrition insecure through incapacitation of the most productive household members, decreased household economic capacity, decreased household agricultural output, and increased caregiver burden [6], consequently posing higher stunting risk to children under 5 years resident in those households. Moreover, HIV infected children under five exhibit poor growth and metabolism and are at higher risk of recurrent morbidity of malnutrition related diseases which further depletes their valuable nutrients [7]. In that matrix, household coping strategies in abating stunting in children under 5 years depends on a complex of economic, health and socio-cultural characteristics of the household including gender of the household head [8]. Specifically, the situation becomes even more critical if the household head is female due to socio-cultural and economic factors which place women at a disadvantage in terms of both contracting HIV and affording nutritionally rich diets for their families [9].

Studies that use a large-scale dataset to establish the link between household gender heterogeneity and HIV status on stunting in Sub-Saharan Africa remain scant. With the current global increase in the drive to use nutritional interventions to combat stunting, in that respect the interlink between household HIV status and gender of the household head provides a unique challenge to countries in sub-Saharan Africa. Therefore, this study contributes to the ongoing discussions on the basis of usage of a large-scale dataset of 13,854 households in Zimbabwe, one of the countries most affected by the HIV epidemic. Specifically, the study seeks to answer three research questions: (i) what is the association between gender of the household head and household HIV/AIDS status?, (ii) secondly, are households with people living with HIV/AIDS more likely to have a stunted child under 5 years old than those without?, and (iii) is there gender heterogeneity in the impact of household HIV/AIDS status on household propensity to have stunted children?. Identification of the impact of HIV/AIDS on the stunting in children under 5 years is however confounded by self-selection bias associated with household HIV/AIDS status. We mitigate the self-selection bias using propensity score matching (PSM) techniques.

The study offers three major findings. Firstly, there is no significant association between gender of the household head and household HIV/AIDS status. Secondly, households that have at least one HIV positive member are more likely to have a stunted child under 5 years. Lastly, female headed households with at least one HIV positive member are more likely to have a stunted child under years compared to male headed household with similar HIV/AIDS status.

The rest of the study is organized as follows; Methods address the definition of variables, model specification and the estimation procedure employed. Results presents the results and discussion of the results. Discussion summarizes the study and provides policy recommendations.

## Methods

### Sample size

This paper is based on secondary analysis of existing data from the 2019 Zimbabwe Rural and Urban Livelihoods Surveys. The annual cross-section surveys are conducted by the Zimbabwe Vulnerability Assessment Committee (ZimVAC) which pools together the Zimbabwean government, UN agencies and non-governmental organizations. The total sample size comprises of 13,854 households both urban and rural. The gender disaggregation of the total sample comprises 6084 female headed households and 7, 770 male headed households. A total of 1, 804 households have at least one HIV positive member for which 58% of those are female headed.

### Measurement of key variables

#### Stunting in children under 5 years old

To measure stunting, the study employed the WHO methodology as described in De Onis et al. [10]. The WHO Anthro software version 3.2.2 was used to analyse Anthropometric HAZ index Z-score. The proportion of stunted children under 5 years old in the household *i* (outcome variable) was thus measured as:

$$ {Y}_i=\frac{\boldsymbol{Number}\ \boldsymbol{of}\ \boldsymbol{children}\ \boldsymbol{under}\ 5\ \boldsymbol{years}\ \boldsymbol{with}\ \mathrm{HAZ}<2\ \boldsymbol{in}\ \boldsymbol{household}\ \mathrm{i}}{\boldsymbol{Number}\ \boldsymbol{of}\ \boldsymbol{children}\ \boldsymbol{under}\ 5\ \boldsymbol{years}\ \boldsymbol{in}\ \boldsymbol{household}\ \mathrm{i}} $$

#### Household HIV status

To measure household HIV/AIDS status, HIV/AIDS statuses for every member of the household were recorded. If at least one member of household *i* was HIV positive, then household *i* was deemed to be affected by HIV/AIDS.

### Econometric specification

#### The impacts of gender and on HIV status

To test Hypotheses 1 of this study, which speaks to the impact of gender of the household head on the probability of the household having an HIV positive member, we employed binary response models as follows:

1 $$ {HIV}_i=a+{\beta}_1{\boldsymbol{Female}}_i+{X}_i^{\prime }y+{\varepsilon}_i $$

*HIV*_*i*_ is the household HIV status which assumes a value of 1 if at least one member of the household is HIV positive and 0 otherwise. *Female*_*i*_ is a dummy variable indicating the gender of the household head, which takes the value of 1 if the household head is female and 0 otherwise. ***X***_***i***_ is a vector of the household background characteristics.

#### The impact of HIV status on stunting

We estimated impact of household HIV status on the proportion of stunted children under 5 years in the household using Propensity Score Matching (PSM) method. Since the causal variable of interest *HIV*_*i*_ is a treatment dummy, and the model is confounded by incomplete information emanating from self-selection into treatment (*HIV*_*i*_ =1) and control (*HIV*_*i*_ =0), PSM is ideal. Propensity score matching circumvents the problem of self-selection by mimicking randomisation of treatment and creating a credible counterfactual group [11]. However, one of the limitations of PSM is that it assumes that any covariate has a linear effect on stunting [12]. In this study, we used nearest neighbour matching technique which chooses an individual from the comparison group for treated individual that is closest in terms of propensity score. We estimate the average treatment effect on the treated that provides the impact of household head gender on the proportion of stunted children under 5 years within the household and household HIV status on the proportion of stunted children under 5 years within the household.

## Results

### Descriptive analysis

#### Background characteristics of sample households

Table 1 displays the differences in the background characteristics of the households by the HIV status of the household. The table shows that 1804 (13%) households had at least one HIV positive household member whereas 12,050 (87%) of the households did not have an HIV positive household member. In addition, Table 1 shows that before controlling for observed confounding variables, households that have at least one HIV positive member are more likely to be female headed than their counterparts that do not have an HIV positive member. The difference in respective proportions of 11.8% is statistically valid at the 1% level of significance. The results on other variables in Table 1 shows that comparing, households that have at least one member who is HIV positive and those without, there are marked differences in marital status, education, income among other variables. The result therefore points to self-selection bias associated with household HIV status. These results mirror those from the study of Adeyemi [13] where the results showed socio-economic characteristics such as marital status, education and income level as some of the determinants of sexually transmitted infections.

**Table 1:** Background characteristics by HIV/AIDS status of the household

		Household has an HIV positive member?				Difference in means [Y – N]
		Yes [Y]		No [N]		
		Mean	S. D	Mean	S. D	
Observations (%)		1804	(13%)	12,050	(87%)	
Household head is female		0.580	0.494	0.462	0.499	0.118^a^
Household head age (Years)		45.555	15.049	38.854	14.577	6.701^a^
Marital status:	Married living together	0.589	0.492	0.760	0.427	−0.170^a^
	Married living apart	0.061	0.239	0.077	0.267	−0.016^a^
	Divorced/separated	0.097	0.296	0.050	0.218	0.047^a^
	Widow/widower	0.213	0.409	0.080	0.271	0.133^a^
Household head education level	Primary level	0.377	0.485	0.261	0.439	0.116^a^
	ZJC level	0.169	0.375	0.122	0.327	0.047^a^
	O′ level	0.302	0.459	0.453	0.498	−0.150^a^
	A’ level	0.013	0.113	0.034	0.181	−0.021^a^
	Diploma/Certificate after primary	0.002	0.040	0.005	0.067	−0.003^a^
	Diploma/Certificate after secondary	0.009	0.095	0.030	0.171	−0.021^a^
	Graduate/Post-Graduate	0.006	0.077	0.020	0.139	−0.014^a^
Household head religion:	Roman Catholic	0.080	0.272	0.077	0.266	0.004
	Protestant	0.084	0.277	0.087	0.283	−0.004
	Pentecostal	0.191	0.393	0.214	0.410	−0.023^b^
	Apostolic Sect	0.270	0.444	0.296	0.456	−0.026^b^
	Zion	0.101	0.301	0.074	0.262	0.027^a^
	Traditional	0.024	0.154	0.017	0.130	0.007^c^
	No religion	0.139	0.346	0.121	0.326	0.018^c^
Household size		5.811	2.292	5.168	1.973	0.643^a^
Monthly income		356.347	746.649	623.581	1315.069	−267.23^a^
Mentally ill household members		0.185	0.520	0.115	0.391	0.070^a^
Household members with mother alive		2.867	1.590	2.529	1.435	0.338^a^
Household members with father alive		2.420	1.515	2.365	1.395	0.055
Household is located in rural areas		0.671	0.470	0.515	0.500	0.156^a^
Province	Bulawayo	0.025	0.156	0.023	0.150	0.002
	Manicaland	0.067	0.250	0.097	0.296	−0.030^a^
	Mash Central	0.094	0.292	0.117	0.321	−0.023^a^
	Mash East	0.115	0.319	0.153	0.360	−0.038^a^
	Mash West	0.126	0.332	0.139	0.346	−0.013
	Mat North	0.129	0.335	0.074	0.262	0.054^a^
	Mat South	0.142	0.349	0.092	0.289	0.050^a^
	Midlands	0.158	0.365	0.135	0.342	0.023^b^
	Masvingo	0.085	0.279	0.085	0.279	0.000
	Harare	0.060	0.237	0.085	0.279	−0.025^a^

Notes: The last column shows the results of two-tailed t-test for the difference in the means. ^a^, ^b^, and ^c^ indicate the 1, 5, and 10% levels of significance

#### Stunting in children under 5 years by household HIV status

Table 2 shows that households that have a member who is HIV positive are more likely to have a stunted child under 5 years than those households without an HIV positive member before controlling for observed covariates. At least 27.7% of households that have an HIV positive member have at least one stunted child versus 23.6% of the households that do not have an HIV positive member. The difference in proportion of 4.1% is statistically valid at the 1% level of significance.

**Table 2:** Proportion of households with stunted children by household HIV status

	Status	Mean	S. D	N
Household has an HIV positive member?	Yes [Y]	0.277	0.447	1804
	No [N]	0.236	0.424	12,050
Difference in means [Y – N]		0.041^a,b,c^		

Notes: The last row shows the results of two-tailed t-test for the difference in the means. ^a^, ^b^, and ^c^ indicate the 1, 5, and 10% levels of significance

### Econometric estimation results

#### Association of gender and HIV status of the households

Columns (I) and (II) of Table 3 displays no statistically significant association of the gender of the household head and the HIV status of the household. This finding is consistent with Hypothesis 1 of this study which notes that there are no gender differences in the household HIV status. However, this result is in contrast with results from the study of Shisana et al. [14], which revealed that in South Africa, female heads of households were significantly more likely to be infected with HIV than their male counterparts (17.9% vs. 13.1%). The results in Table 3 (Column I) however show that household head marital status, education, as well as household income are associated with changes in the HIV status of the household. These results are similar to trends reported by Shisana et al. [14], Curry et al. [15] and Ndirangu et al. [16].

**Table 3:** Household head gender association with household HIV/AIDS status

	Household has an HIV positive member	
VARIABLES	Logit	Probit
	(I)	(II)
Household head is female	0.0790	0.00992
	(0.0855)	(0.00737)
Household head age [Years]	0.0141***	0.00167***
	(0.00233)	(0.000289)
Married living together	−0.712***	− 0.0729***
	(0.176)	(0.0226)
Married living apart	−0.734***	−0.0756***
	(0.204)	(0.0243)
Primary level	0.223**	0.0262*
	(0.102)	(0.0148)
ZJC level	0.454***	0.0506***
	(0.121)	(0.0168)
O′ level	0.0112	0.00546
	(0.120)	(0.0150)
A’ level	−0.294	−0.00609
	(0.240)	(0.0187)
Diploma/Certificate after primary	−0.727	−0.0462
	(0.630)	(0.0349)
Diploma/Certificate after secondary	−0.739***	−0.0351*
	(0.283)	(0.0182)
Graduate/Post-Graduate	−0.704**	−0.0299
	(0.343)	(0.0195)
Protestant	0.0648	0.00669
	(0.145)	(0.0147)
Pentecostal	0.164	0.0166
	(0.124)	(0.0126)
Apostolic Sect	0.0144	0.00164
	(0.120)	(0.0124)
Zion	0.184	0.0217
	(0.142)	(0.0161)
Islam	−0.0501	−0.00259
	(0.370)	(0.0349)
No religion	0.351***	0.0373***
	(0.135)	(0.0145)
Household size	0.105***	0.0136***
	(0.0239)	(0.00313)
Monthly income	−0.0938***	−0.00994***
	(0.0250)	(0.00266)
Mentally ill household members	0.0976	0.0153
	(0.0666)	(0.00976)
Household members with mother alive	0.0648*	0.0140**
	(0.0392)	(0.00621)
Household members with father alive	−0.127***	−0.0231***
	(0.0334)	(0.00564)
Household is located in rural areas	0.174*	0.0177*
	(0.0903)	(0.00910)
Constant	−2.340***	0.0739**
	(0.325)	(0.0365)
Observations	12,162	12,162
R-squared		0.069

Robust standard errors in parentheses

*** *p* < 0.01, ** *p* < 0.05, * *p* < 0.1

#### Impact of household HIV/AIDS status on stunting in children under 5 years

Table 4 shows the Propensity Score Matching (PSM) estimates of the average treatment effects of household HIV positive status on the probability of the household having at least one stunted child under 5 years. The results indicate that if the household has at least one HIV positive member, the probability that the household has a stunted child under 5 years increases by 4.55% at the 1% level of significance, ceteris paribus. These results in Tables 3 and 4 are therefore consistent with Hypothesis 2 of this study, which notes that households that have an HIV positive member are associated with higher probability of having stunted children.

**Table 4:** PSM estimates of the impact of household HIV/AIDS on stunting in children under five years

Average treatment effect (ATE) of household HIV positive status on probability of having stunted child	0.0455***
	(0.0139)
Observations	11,763

Robust standard errors in parentheses

*** *p* < 0.01, ** *p* < 0.05, * *p* < 0.1

#### Gender heterogeneity treatment effects of HIV status on stunting in children under 5 years

Table 5 shows the PSM estimates of gender heterogeneity and the impact of household HIV positive status on the probability that the household has a stunted child under 5 years. The results indicate that female headed households that have an HIV positive member have a higher probability of having a stunted child than their male counterparts. Column (I) of Table 5 shows that female headed households that have an HIV positive member have 6.66% likelihood of having a stunted child versus the 3.26% for the male headed households displayed in Column (II). These results therefore affirm Hypothesis 3 of this study, that the impact of HIV positive status on the proportion of stunted children in the household is higher in female headed households, *vis-à-vis* male headed households.

**Table 5:** PSM estimates of gender heterogenic treatment effects of household HIV status on stunting in children under five years

	Household head is female?	
VARIABLES	Yes	No
	(I)	(II)
ATE of household HIV positive status on probability of having stunted child	0.0666***	0.0326*
	(0.0215)	(0.0186)
Observations	4692	7071

Robust standard errors in parentheses *** *p* < 0.01, ** *p* < 0.05, * *p* < 0.1

## Discussion

The results presented in Tables 2, 3, 4 and 5 are in concurrence with previous studies. A study on association between HIV/AIDS and nutritional status of children under 5 years of age conducted in Kenya [16] found that children living in HIV/AIDS affected households had a significantly higher prevalence of stunting (25.5%) than children in unaffected households (9.1%). Results from a secondary analysis of the Demographic and Health Survey (DHS) data collected from 18 countries in sub-Saharan Africa [17] also reviewed similar trend, where children from HIV/AIDS affected households were significantly more likely to be stunted compared to their counterparts of similar demographic and socio-economic background but from households not affected by HIV/AIDS.

The observed results in this study, that children from HIV/AIDS affected households are highly likely to be stunted compared to their counterparts from households not affected by HIV/AIDS, might be attributed to the fact that resources that would have been directed to providing adequate nourishment for the children in HIV-affected households being diverted to manage the effects of HIV/AIDS, such as paying for healthcare for sick family members, including the children themselves [18–20]. This finding is supported by previous findings, e.g. Ndirangu et al. [16], Kimani-Murage et al. [21] and Sunguya et al. [22], which indicate a significantly higher degree of stunting in children from HIV/AIDS affected households than children in unaffected households. The studies concluded that HIV is an independent modifiable risk factor for poor nutritional outcomes (such as stunting) and makes a significant contribution to nutritional outcomes at the individual level.

It is also probable that the adults responsible for providing for the family were less productive because of illness and therefore provided less food for the family [16]. This also agrees with findings from [23], which revealed that the productivity of HIV-affected household members is reduced, affecting household’s affordability of good quality and nutritious food. More so, a study by Chege et al. [18] reported that most household headed by females have less income compared to male-headed households which is likely to impact on household food and nutrition security.

## Conclusion

Overall, the results presented in this study provide evidence of a higher risk of stunting among children from households affected by HIV/AIDS. The results confirm the three assumptions postulated in this study that; (i) female headed households are no more likely to be affected by HIV/AIDS, (ii) household HIV/AIDS status adversely affects incidence of stunting in children under 5 years, and (iii) the impact of household HIV positive status on the incidence of stunting in children under 5 years is higher in female headed households compared to male headed households.

Furthermore, the results reviewed that education is associated with less HIV infection. Overall, the findings have important implications for policy and programme efforts towards improved integration of HIV/AIDS and child nutrition services in affected communities and households. In particular, children from HIV/AIDS affected households deserve special attention. In addition, there is need for effective and tailored nutrition-sensitive and specific interventions using multisectoral approaches should be considered to address these important determinants.

## Data Availability

The datasets analysed in the current study are available from the Food and Nutrition Council of Zimbabwe (FNC) but restrictions apply to the availability of these data, which were used under a Memorandum of Understanding for the current study, and so are not publicly available. Data are however available from the authors upon reasonable request and with permission of FNC.

## Abbreviations

AIDS: Acquired Immunodeficiency Syndrome
DHS: Demographic and Health Survey
HIV: Human Immunodeficiency Virus
PLWHIV: People Leaving with HIV
PSM: Propensity Score Matching
WHO: World Health Organisation
ZimVAC: Zimbabwe Vulnerability Assessment Committee
